# Brain-targeted intranasal delivery of protein-based gene therapy for treatment of ischemic stroke

**DOI:** 10.7150/thno.98088

**Published:** 2024-08-12

**Authors:** Jee-Yeon Ryu, Christian Cerecedo-Lopez, Hongkuan Yang, Ilhwan Ryu, Rose Du

**Affiliations:** 1 Department of Neurosurgery, Brigham and Women's Hospital, Harvard Medical School, Boston, MA 02115, United States.; 2 Department of Surgery, Valley Baptist Medical Center, University of Texas Rio Grande Valley, Harlingen, TX 78550, United States.; 3 Department of Neurosurgery, Tongji Hospital, Tongji Medical College, Huazhong University of Science and Technology, Wuhan 430030, China.; 4 Department of Chemistry, Kookmin University, Seoul 02707, South Korea.; 5 Cooperative Center for Research Facilities, Kookmin University, Seoul 02707, South Korea.

**Keywords:** Intranasal Delivery, Ischemic Stroke, CRISPR/dCas9, Nanoparticles, Gene Editing

## Abstract

Gene therapy using a protein-based CRISPR system in the brain has practical limitations due to current delivery systems, especially in the presence of arterial occlusion. To overcome these obstacles and improve stability, we designed a system for intranasal administration of gene therapy for the treatment of ischemic stroke.

**Methods:** Nanoparticles containing the protein-based CRISPR/dCas9 system targeting *Sirt1* were delivered intranasally to the brain in a mouse model of ischemic stroke. The CRISPR/dCas9 system was encapsulated with calcium phosphate (CaP) nanoparticles to prevent them from being degraded. They were then conjugated with β-hydroxybutyrates (bHb) to target monocarboxylic acid transporter 1 (MCT1) in nasal epithelial cells to facilitate their transfer into the brain.

**Results:** Human nasal epithelial cells were shown to uptake and transfer nanoparticles to human brain endothelial cells with high efficiency *in vitro*. The intranasal administration of the dCas9/CaP/PEI-PEG-bHb nanoparticles in mice effectively upregulated the target gene, *Sirt1*, in the brain, decreased cerebral edema and increased survival after permanent middle cerebral artery occlusion. Additionally, we observed no significant *in vivo* toxicity associated with intranasal administration of the nanoparticles, highlighting the safety of this approach.

**Conclusion:** This study demonstrates that the proposed protein-based CRISPR-dCas9 system targeting neuroprotective genes in general, and *SIRT1* in particular, can be a potential novel therapy for acute ischemic stroke.

## Introduction

Stroke is the second most common cause of death in the world and ischemic stroke accounts for up to about 87% of all stroke cases 1-3. Current treatment of acute ischemic stroke from large vessel occlusion are thrombolysis and thrombectomy. However, only 12.5-25% of patients with large vessel occlusion undergo thrombectomies 4. Among patients with acute ischemic stroke, fewer than 5% receive intravenous thrombolysis globally 5 and 12% undergo thrombolysis and/or thrombectomy in the United States 6. The high percentage of patients that do not undergo thrombolysis or thrombectomy is a result of the highly time-sensitive requirements and the lack of access to a stroke center. Efforts to develop new drugs for ischemic stroke beyond those for thrombolysis have been unsuccessful so far 7, 8. Although there are many reasons for the failure to develop new effective therapies for treating ischemic stroke, a major obstacle is the ability to deliver therapeutics when the area affected has limited perfusion. Even in areas that are perfused and may be affected by the secondary effects of infarction, the delivery of drugs is limited by the blood-brain barrier (BBB) 9, 10. Therefore, many studies in recent years have reported the use of intranasal drug administrations to directly target the brain and bypass the BBB 11-13. Monocarboxylic acid transporter 1 (MCT1) is expressed in nasal epithelial cells, and their substrate is known as a β-hydroxybutyrate (bHb). It has been reported that nanoparticles that were decorated by bHb can be transported via human nasal epithelial cells (HNEpCs) to reach the brain 14-16.

Silent information regulator 2 homologue 1 (SIRT1) is highly widespread in the brain, and has been identified to play key roles in controlling neuroendocrine function and protecting against cerebral ischemia 17, 18. *Sirt*^-/-^ mice showed larger infarct volumes in permanent middle cerebral artery occlusion (pMCAO) compared to their control groups 19, 20. In contrast, SIRT1-overexpressing mice had less hippocampal damage in bilateral common carotid artery occlusion than healthy control groups 21, 22. These studies provide evidence that the regulation of SIRT1 can protect against ischemic injury.

CRISPR/Cas9 is an efficient gene editing tool that generates insertions or deletions (indels) in DNA 23-25. The Cas9 protein has recently been repurposed as nuclease dead Cas9 (dCas9), which can regulate gene expression by fusing transcriptional activators (such as p65 and VP64) without creating DNA double-strand breaks 26, 27. These dCas9 systems have been reported in genetic engineering, gene therapy, and the treatment of various diseases 28-31. For delivering the dCas9 system, a protein-based CRISPR system offers several advantages, such as safety, lower off-target effects, and enhanced gene editing efficiency compared to DNA or mRNA delivery formats 32, 33. However, protein-based methods can be easily degraded by proteases or blood cells, and they are generally challenging to deliver using viral vectors due to their large size 34-36. These challenges have proven especially difficult for delivery to the brain *in vivo* 37.

Calcium phosphate (CaP) nanoparticles are widely used as nonviral gene delivery systems for gene therapy due to low immunotoxicity, biocompatibility, and high affinity for binding with nucleic acids 38, 39. The mineralization of Cas9 proteins by CaP efficiently increases the protein stability, and successfully achieved gene editing in cells 40. However, CaP nanoparticles have low rates of delivery which presents a barrier to their use *in vivo*. To solve this problem, several strategies have been used to control the CaP nanoparticles, such as polymer stabilization using polyethylene glycol (PEG) 41-43. PEG-mediated nanoparticles have been used successfully to enable delivery of proteins in clinical trials 44-46. Therefore, we proposed a novel method to design and synthesize a nanoparticle composed of mineralized-protein-based dCas9-VP64 system with CaP coated with PEG, and PEG residues conjugated to bHb.

Here, we show that intranasal administration is effective for delivering novel nanoparticles for protein-based gene therapy to the ischemic brain with large vessel occlusion. We constructed nanoparticles conjugated with bHb for intranasal administration, and loaded the nanoparticles with a protein-based dCas9-VP64 system that targets *Sirt1*. The nanoparticles successfully delivered the dCas9-VP64 system through the nose to the brain via bHb, and *Sirt1* gene was efficiently upregulated by the protein-based dCas9-VP64. As a proof-of-concept study, this study provides a new strategy for delivering the protein-based gene therapy to the brain that bypasses the BBB and demonstrates that *SIRT1* could be an attractive candidate for the treatment of ischemic stroke.

## Methods

### *In vitro* transcription of sgRNAs and purification of the dCas9-VP64 protein

*In vitro* sgRNA transcriptions were generated from a dsDNA template containing the protospacer and the trans-activating crRNA (tracRNA) sequence for targeting both *SIRT1* for human and *Sirt1* for mouse. The sgRNA sequences were designed or selected using online tools provided by Feng Zhang's laboratory at MIT to uniquely and robustly activate transcription of the endogenous gene when used with the CRISPR/dCas9 activator system. The sgRNAs sequences were also checked by CHOPCHOP and CRISPR RGEN Tools. The following primers were used for the PCR amplification: sgRNA1: GGGCCGGACCACAACACGCC, sgRNA2: GTGTTGTGGTCCGGCCCGCG, and sgRNA3: CCACACACGCCGGGTCACG. The templates of sgRNAs were incubated with T7 RNA polymerase (NEB) in reaction buffer [40 mM Tris-HCl (pH 8.0), 6 mM MgCl_2_, 10 mM DTT, 10 mM NaCl, NTP, RNase inhibitor] at 37°C for 4 h. *In vitro* transcribed RNA was incubated with DNase I (Takara) to remove the template DNA, and was extracted with phenol:chloroform:isoamyl alcohol (25:24:1, v/v/v), saturated with 10 mM Tris (pH 8.0) and 1 mM EDTA. For purification of dCas9-VP64 proteins, *Escherichia coli* (NEB) was transformed with a pET-dCas9-VP64 vector encoded His-tag at the N-terminus (Addgene). The cells were incubated with 0.5 mM isopropyl-β-D-1-thiogalactopyranoside (IPTG, Sigma) at 28°C to facilitate dCas9-VP64 protein expression and lysed in a lysis buffer [20 mM Tris-Cl (pH 8.0), 300 mM NaCl, 20 mM imidazole, 1 × protease inhibitor cocktail, 1 mg/mL lysozyme] by sonication. The lysate was obtained after centrifugation at 20,000×g for 20 min at 4°C, and the soluble fraction was incubated with a Ni-NTA agarose resin (Qiagen) to capture the His-tagged dCas9-VP64 proteins. The column-bound dCas9-VP64 proteins were eluted using an elution buffer [20 mM Tris-Cl (pH 8.0), 300 mM NaCl, 300 mM imidazole] and then dialyzed into a storage buffer [50 mM Tris-HCl (pH 8.0), 200 mM NaCl, 0.1 mM EDTA, 1 mM DTT, 0.5 mM PMSF, 20% glycerol]. The purified dCas9-VP64 proteins were quantified using bicinchoninic acid (BCA) assay (Pierce Biotechnology) and were analyzed by SDS-PAGE and detected by Coomassie blue. To prepare dCas9-VP64/sgRNA, we mixed dCas9-VP64 proteins with sgRNAs at a ratio of 1:3 molar ratio.

### Preparation of dCas9/CaP/PEI-PEG-bHb

To encapsulate dCas9-VP64/sgRNA into the core of a nanoparticle, 120 mM Na_2_HPO_4_ and 0.1 M NaOH were dissolved in 4 mL PBS, and added to the prepared 1mL dCas9-VP64/sgRNA containing 100 μg of dCas9 proteins and 59.3 μg of gRNA molecules. The mixture was stirred at 24°C for 1 hour and 100 mM CaCl_2_ in 5 mL PBS was added to the mixture. The dCas9/CaP particles were washed three times with PBS using a centrifuge at 12,000×g for 20 min, and resuspended in 1mL PBS. 0.5 mM Polyethylenimine-Polyethylene glycol (PEI-PEG, Sigma) in 4 mL PBS was added to the 1mL dCas9/CaP, and stirred at room temperature for 2 hours to coat the surface of the dCas9/CaP. The dCas9/CaP/PEI-PEG was washed three times with PBS using a centrifuge at 12,000×g for 20 min, and resuspended in 1mL PBS.

To conjugate β-hydroxybutyric acid (bHb) onto the surface of the dCas9/CaP/PEI-PEG, 0.5 mM amine-terminated PEGs (Sigma) in 1.8 mL PBS were mixed with 200 μl conjugation buffer [0.1M 2-[N-morpholino]ethane sulfonic acid (MES, pH 4.5-5), Thermo Scientific]. The mixture was added to 0.3 mM bHb terminated with carboxylic acid groups in 2mL PBS, and 1mL 1-Ethyl-3-[3-dimethylaminopropyl]carbodiimide hydrochloride (EDC) solution in the last step, and reacted for 3 hours at room temperature. The bHbs conjugated with PEGs (PEG-bHbs) were purified using desalting columns (Thermo Scientific), and dissolved in 4 mL PBS. The 4mL of PEG-bHbs were then mixed with 1mL dCas9/CaP/PEI-PEG solution (containing 100 μg of dCas9 proteins and 59.3 μg of gRNA molecules) at room temperature for 2 hours. The dCas9/CaP/PEI-PEG-bHb was washed three times with PBS using a centrifuge at 12,000×g for 20 min, and resuspended in 1 mL PBS. The final dCas9/CaP/PEI-PEG-bHb solution was diluted 1/100 for cell experiments, and 1/10 for mouse experiments.

### Characterization of the nanoparticles

The morphologies of the nanoparticles were imaged using field emission scanning electron microscopy (FE-SEM, JSM-7401F, JEOL), and high-resolution transmission electron microscopy (HR-TEM, AVANCE III, JEOL). The X-ray photoelectron spectroscopy (XPS) measurements were carried out using a K-alpha system (Thermo Scientific).

### Cell culture and co-culture

Vascular smooth muscle cells (vSMCs) were cultured in smooth muscle cell medium (ScienCell) supplemented with 10% FBS, 1% smooth muscle cell growth supplement (ScienCell), and 1% penicillin-streptomycin. BT474 and human brain endothelial cells (HBECs) were cultured in DMEM medium (Gibco) and DMEM:F12 (Gibco) supplemented with 10% FBS and 1% penicillin-streptomycin. Human nasal epithelial cells (HNEpCs) were cultured in airway epithelial cell growth medium (PromoCell) supplemented with epithelial cell growth mixture (PromoCell) and 1% penicillin-streptomycin. All cells were maintained in a humidified 37°C incubator at 5% CO_2_. For co-culture, HNEpCs were seeded onto transwell inserts (pore size: 0.4 μm, Corning) and after 1 day, treated with each nanoparticle (containing 1 μg of dCas9 proteins and 0.6 μg of gRNA molecules), including dCas9/CaP, dCas9/CaP/PEI-PEG-bHb without dCas9 (Empty/CaP/PEI-PEG), dCas9/CaP/PEI-PEG with or without bHb for 2 hours. The HNEpCs were transferred to the wells with HBECs present at the bottom of the wells. The HBECs were analyzed by a confocal laser scanning microscopy (CLSM) after 24 hours.

### Mouse experiments

*Animals*. All experiments were approved by the Institutional Animal Care and Use Committee of Brigham and Women's Hospital (BWH, Protocol# 2016N000291), complied with the Public Health and Services Policy, the Animal Welfare Regulations, and BWH Institutional Animal Care and Use Committee (IACUC) policies and guidelines. Male BALB/cJ mice were purchased and were housed in a 12h light/dark cycle, temperature-controlled room. Animals were divided into groups in a randomized fashion, and experiments were performed in a blinded manner.

*pMCAO model*. To induce permanent MCAO (pMCAO), all mice were anesthetized, a vertical midline cervical incision was made, and the right common carotid artery (CCA) was exposed and dissected. The external carotid artery (ECA) and internal carotid artery (ICA) were then exposed. After the ECA was ligated, a monofilament nylon suture was inserted through the ICA to the middle cerebral artery (MCA). Insertion was stopped when resistance was felt. After surgery, the incision was closed with sutures. All mice recovered from anesthesia in a 37°C container. Sham surgery included the exposure of the common, external, and internal carotid arteries; no MCA occlusion occurred in the sham group. We utilized a combination of methods to confirm stroke development in the mice after surgery. We evaluated motor function, balance, and sensory function, and measured MCA blood flow using a laser Doppler flowmeter. We implemented inclusion and exclusion criteria to ensure data quality and animal well-being. Animals were included if they fell within a weight range (25.0 ± 1.4), exhibited no pre-existing health conditions, and underwent successful MCAO surgery with confirmed cerebral blood flow reduction. Conversely, animals were excluded if they experienced complications during surgery, displayed significant weight loss or health problems post-surgery, or had inconsistent behavioral test results suggesting potential pre-existing issues.

### Delivery of the nanoparticles

The mice were randomly divided into five groups for delivery of nanoparticles (each group had ten mice). Each group was treated with 100 μl of each nanoparticle (containing 10 μg of dCas9 protein and 6 μg of gRNA molecules), including dCas9-VP64, dCas9/CaP, dCas9/CaP/PEI-PEG, dCas9/CaP/PEI-PEG-bHb without dCas9-VP64 (Empty/CaP/PEI-PEG-bHb), and dCas9/CaP/PEI-PEG-bHb after pMCAO. The dose and amount of the nanoparticles delivered intranasally was determined based on previous studies 47-49. All nanoparticles were intranasally delivered to the mice 3 hours after surgery. As control groups, we used two groups: control mice (non-surgery and non-treated) and pMCAO model mice (non-treated). Body weight was measured every four days.

### Reverse transcription - quantitative PCR (RT-qPCR)

Total RNA was isolated using the RNeasy RNA isolation kit (Qiagen). cDNA was synthesized using 1 μg of total RNA from harvested mouse tissues with the Maxima cDNA Synthesis Kit for RT-qPCR (Thermo Scientific). RT-qPCR was performed using SYBR Green Master Mix (Applied Biosystems) on the AB step one plus real-time PCR system (Applied Biosystems). The primer sequences for amplification were:

SIRT1_F: 5'-GCAGATTAGTAGGCGGCTTG-3',

SIRT1_R: 5'-TCTGGCATGTCCCACTATCA-3',

Sirt1_F: 5'-GATCCTTCAGTGTCATGGTT-3',

Sirt1_R: 5'-GAAGACAATCTCTGGCTTCA-3',

Bcl2_F: 5'-CTCAGGCTGGAAGGAGAAGAT-3',

Bcl2_R: 5'-AAGCTGTCACAGAGGGGCTAC-3',

Bax_F: 5'-TGGAGATGAACTGGACAGCAATAT-3',

Bax_R: 5'-GCAAAGTAGAAGAGGGCAACCAC-3',

Mmp9_F: 5'-TGAATCAGCTGGCTTTTGTG-3',

Mmp9_R: 5'-ACCTTCCAGTAGGGGCAACT-3',

Parp_F: 5'-AGGCCCTAAAGGCTCAGAAT-3',

Parp_R: 5'-CTAGGTTTCTGTGTCTTGAC-3'.

Real-time PCR conditions were as follows: a reaction for 4 min at 95 °C for initial denaturation, followed by 40 cycles of denaturation for 20 s at 95 °C, annealing for 20 s at 60 °C, and extension for 15 s at 72 °C. Finally, a final extension was done for 20 s at 72°C.

### Brain water content (BWC)

To evaluate the effect of the nanoparticles on brain edema, we used the wet/dry weight method. The wet weight of each brain was carefully weighed and recorded. The dry weight was weighed after drying the brain in an oven at 70°C. Brain water content (%) was determined using this formula:

% Brain water content = (Wet weight -Dry weight) / (Wet weight) ×100

### Western blotting

Harvested cells and mouse tissues were lysed in RIPA Lysis and Extraction Buffer (Thermo Scientific) and protein concentration was measured using a BCA assay (Pierce). Total protein (20 μg per well) was separated by SDS-PAGE and transferred to a PDVF membrane. The membrane was incubated with antibodies: Anti-SIRT1, Anti-MCT1, Anti-BCl2, Anti-BAX, Anti-MMP9 and Anti-PARP (Cell Signaling Technology). The membrane was visualized using ImageQuant LAS 500 (GE Healthcare).

### Immunohistochemistry and Immunofluorescence

Brain tissues were dissected from mice and embedded in optimal cutting temperature medium (O.C.T.). The cryosections (thickness: 10 μm) were cut in a freezing cryostat at -20°C. The sections were fixed in ice-methanol for 10 min after having dried on the slide, permeabilized with 0.25 % Triton X-100 (Sigma) at room temperature for 15 min and blocked with blocking solution [0.3 % Triton-X 100, 5 % Normal goat serum in TBS]. For immunohistochemistry (IHC), the sections were incubated with anti-Cas9 (Origene) overnight at 4°C. The sections were then stained with hematoxylin and cover-slipped using a mounting medium. For immunofluorescence (IF), the sections were incubated with anti-Cas9 (Origene), anti-MCT1 or anti-CD31 (Cell Signaling Technology) overnight at 4°C. After three washes with PBS, the sections were incubated for 1 hour at room temperature with a fluorescent secondary antibody (Alexa Fluor 488-conjugated goat anti-mouse or Alexa Fluor 568-conjugated goat anti-mouse, Abcam) in the dark. The sections were mounted with medium (Abcam) containing 4, 6-diamidino-2-pheylindole (DAPI) as a marker for cell nuclei, and images were taken on a Leica TCS-SP5 confocal laser scanning microscope (CLSM).

### Terminal deoxynucleotidyl transferase (TdT)-mediated dUTP nick end labeling (TUNEL) assay

To evaluate apoptotic cells in the brain, TUNEL assay was performed using a TUNEL assay kit (Abcam) according to the manufacturer's instructions. The brain tissue sections were fixed in 4% paraformaldehyde, and were permeabilized in 0.25% Triton X-100 and 0.1% sodium citrate. The sections were incubated with the TUNEL reaction mixture in a humidified atmosphere for 2 h at 37°C in the dark. After being washed with wash buffer three times, the sections were mounted with DAPI mounting medium (Abcam) and analyzed by CLSM.

### Statistical analysis

The statistical significance of the data was determined by using the Student's t-test. Significance among multiple groups were determined by one-way ANOVA or two-way ANOVA following by Tukey's post hoc test. All values were expressed as the mean ± standard deviation. Statistical and graphical analyses were performed with Prism software (GraphPad Software).

## Results

### Strategy for intranasal administration of protein-based dCas9-VP64 delivery system for target gene activation

**Figure 1:**
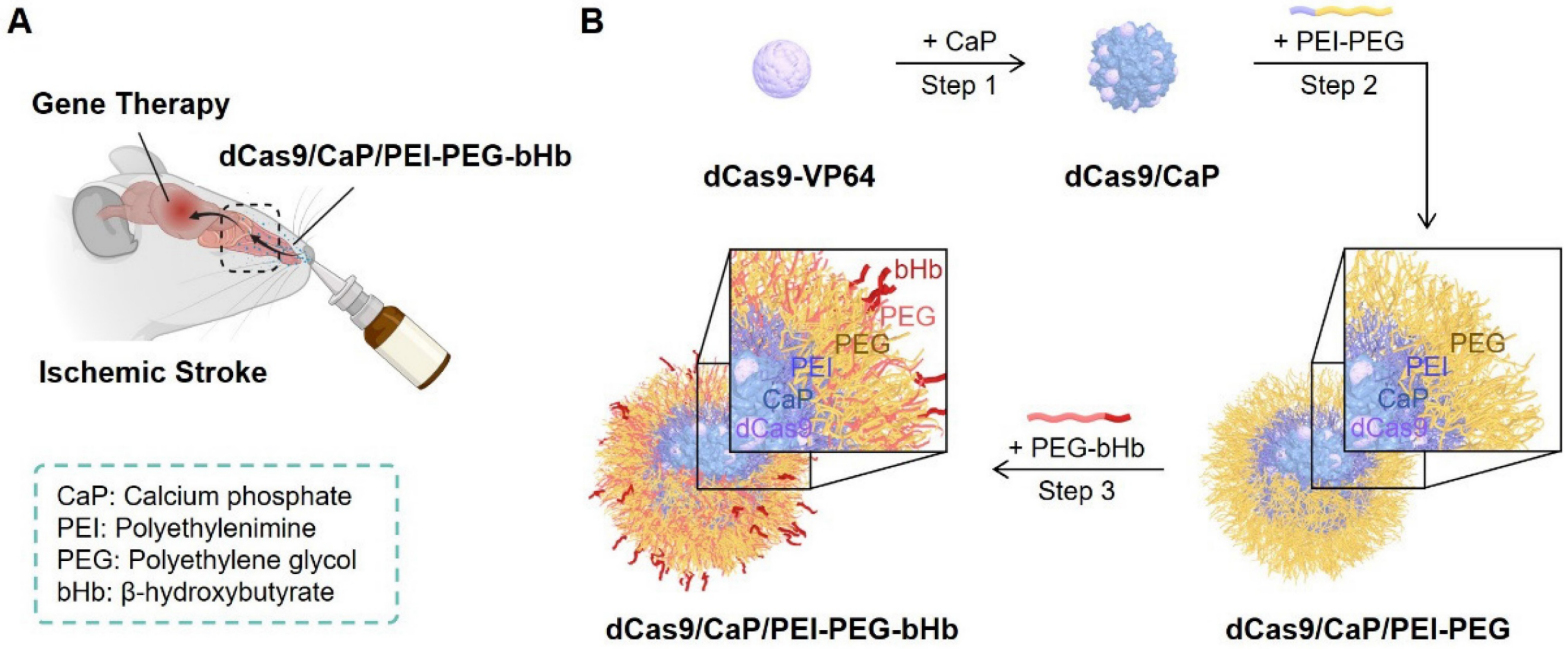
Intranasal administration of dCas9/CaP/PEI-PEG-bHb for treatment of ischemic stroke. **A** Schematic illustration of gene therapy in the brain. Figure created with BioRender.com. **B** Schematic diagram of dCas9/CaP/PEI-PEG-bHb preparation.

The basic concept for intranasal administration of gene therapy for ischemic stroke is illustrated in **Figure 1A**. We developed a new synthetic method to incorporate a mineralized-protein-based dCas9-VP64 system with calcium phosphate (CaP) coated with PEG, and PEG residues conjugated to bHb. When the β-hydroxybutyric acid (bHb)-linked dCas9/CaP/PEI-PEGs are delivered to nose, the bHb can bind with a monocarboxylate transporter 1 (MCT1) in the nasal epithelial cells to transfer the nanoparticles to the brain.

A nuclease dead Cas9 (dCas9) has been extensively explored to fuse with transcriptional activators or repressors (e.g., dCas9-VP64, tripartite activator system (dCas9-VPR), dCas9-Suntag, or synergistic activation mediator (SAM)) to regulate target genes 50-52. However, these systems still exceed the capacity of most common viral vectors (e.g., adeno-associated virus) or vehicles, and also need multiple guide RNAs 53, 54. To solve these issues, we sought to use the protein-based dCas9-VP64 with a single-guide RNA (sgRNA), and develop a delivery system in which the dCas9-VP64 system can be delivered efficiently and safely *in vivo*. As a first step towards preparing the protein-based dCas9-VP64 system, we designed three different sgRNA target sites within the *SIRT1* promoter region using sgRNA design tools for high efficiency and specificity (**Figure S1A**). To deliver the dCas9-VP64 protein, we purified the dCas9-VP64 protein containing a nuclear localization signal (NLS) from *Escherichia coli* (**Figure S1B**). Then we co-transfected each sgRNA and dCas9-VP64 protein in human brain endothelial cells (HBECs) using cationic reagents, and measured the mRNA expression levels of *SIRT1* using reverse transcription-quantitative PCR (RT-qPCR) (**Figure S1C**). We chose the sgRNA1 sequence to deliver the dCas9-VP64 system to the brain via the nasal route as it is the sgRNA with the highest efficiency. These data demonstrate that the dCas9-VP64 protein with sgRNA can induce transcription of the targeted gene.

### Establishment of the dCas9/CaP/PEI-PEG-bHb nanoparticle

**Figure 2:**
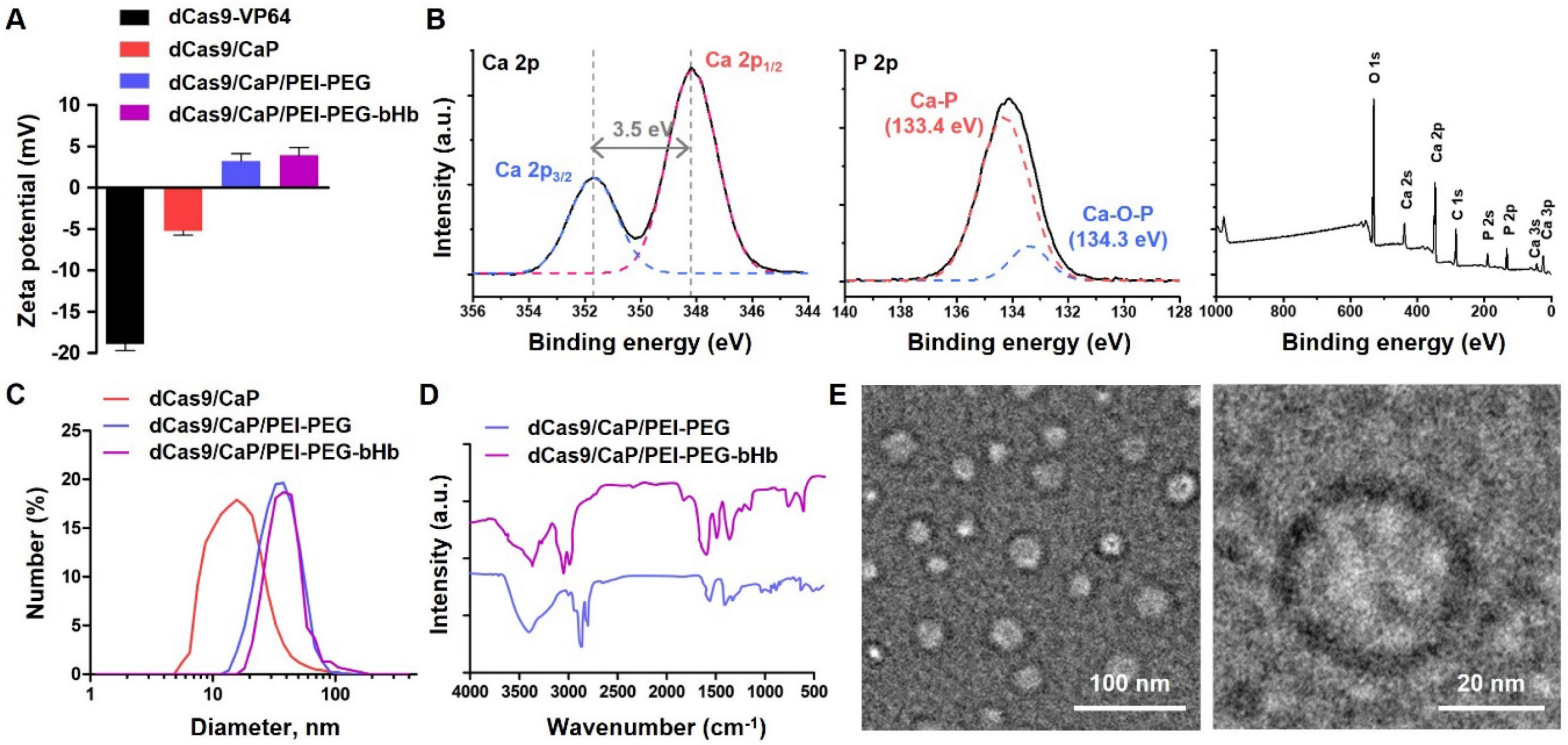
Characterization of dCas9/CaP/PEI-PEG-bHb. **A** Zeta-potentials analysis of the various prepared nanoparticles. **B** X-ray photoelectron spectra of CaP nanoparticles, Ca 2p, P 2p, and XPS survey. **C** Size distributions of the various prepared nanoparticles by dynamic light scattering (DLS). **D** FT-IR spectra of the nanoparticles measured in the fingerprint region (4000-400 cm^-1^). **E** Representative TEM images of dCas9/CaP/PEI-PEG-bHb. Error bars indicate mean ± S.D. (*n* = 3). Abbreviations: dCas9-VP64, dCas9-VP64 protein with sgRNA; dCas9/CaP, dCas9-VP64 protein with sgRNA that was encapsulated with calcium phosphate particles; dCas9/CaP/PEI-PEG; dCas9/CaP that was coated with PEI-PEG; dCas9/CaP/PEI-PEG-bHb; dCas9/CaP/PEI-PEG that was coated with PEG conjugated with β-hydroxybutyric acid (bHb).

To create an intranasal delivery system of the protein-based dCas9-VP64 system, we proceeded to load the dCas9-VP64 proteins with sgRNAs into nanoparticles (**Figure 1B**, step 1). Because the protein-based dCas9-VP64 system has a negative charge (**Figure 2A**), we utilized calcium phosphate (CaP) nanoparticles that were positively charged. The CaP nanoparticles were used for gene therapy due to their strong electrostatic binding affinity with nucleic acids and biocompatibility as non-viral vectors 55. Additionally, calcium phosphates can mineralize with Cas9 ribonucleoprotein (RNP) complexes under physiological conditions 53. We therefore synthesized the dCas9/CaP nanoparticles, and confirmed the sizes and the zeta-potentials of the dCas9/CaP nanoparticles using dynamic light scattering (DLS) (**Figure 2A and 2C**). DLS analysis revealed that the dCas9/CaP nanoparticles had increased zeta-potentials at approximately -5.2 mV. The X-ray photoelectron spectroscopy (XPS) measurements were also investigated to confirm the chemical composition of the CaP nanoparticles (**Figure 2B**). The XPS survey spectra clearly indicated that the CaP nanoparticles consisted of four atoms Ca, P, O, and C. The two peaks from the Ca 2p core level with binding energies of 348.2eV and 351.7eV are consistent with the properties of Ca 2p_1/2_ and Ca 2p_3/2_, respectively. In addition, the main phosphorus contributor, P 2p, can be deconvoluted into two components owing to spin-orbital splitting, 2p_1/2_ and 2p_3/2_ 56, 57. These data showed that dCas9-VP64 proteins were encapsulated inside CaP nanoparticles. However, the dCas9-VP64 proteins still remained in the supernatant after synthesis (**Figure S2**). Thus, we next developed nanoparticles that would incorporate more dCas9-VP64 proteins and still be biocompatible and stable by using polyetherimide (PEI) - polyethylene glycol (PEG) with positive charges (**Figure 1B**, step 2). The sizes and zeta-potentials of the dCas9/CaP/PEI-PEG nanoparticles were approximately 43 nm and + 3.2 mV (**Figure 2A and 2C**), and the dCas9-VP64 proteins no longer remained in the supernatant (**Figure S2**). We next utilized β-hydroxybutyric acid (bHb)-linked PEG (PEG-bHb) to conjugate the bHb onto the surface of the dCas9/CaP/PEI-PEGs (**Figure 1B**, step 3). To conjugate bHb with PEG, bHbs terminated with carboxylic acid groups were mixed with amine-terminated PEGs under 1-Ethyl-3-(3-dimethylaminopropyl)carbodiimide (EDC) reaction (see the Methods for details). The PEG polymer chains of PEG-bHbs can attach to other PEGs on the surface of the dCas9/CaP/PEI-PEGs through covalent bonding. Surface coating of nanoparticles with PEGs has been widely utilized as a good carrier for drugs and nucleic acid therapeutics due to its stabilizing properties 58. We confirmed the formation of bHb on the nanoparticles using FT-IR spectral analysis (**Figure 2D**). The dCas9/CaP/PEI-PEG-bHb was characterized by the increased peaks at 1377 cm^-1^ (OH in plane bending), 1553 cm^-1^ (NH bend) and 1638 cm^-1^ (C=O stretching) 59, 60, indicating the changes on the surface of the dCas9/CaP/PEI-PEG (**Figure 2D**). These data showed the formation of the nanoparticles with bHb conjugate. The morphologies of dCas9/CaP/PEI-PEG-bHb nanoparticles were observed by field emission scanning electron microscopy (FE-SEM) and energy dispersive x-ray spectroscopy (EDX) analysis (**Figure S3A**). Aggregation of the nanoparticles were observed, and CaP and polymer (C, O) were demonstrated in the EDX analysis. The size of the CaP-based nanoparticles was approximately 50 nm on the high-resolution transmission electron microscopy (HR-TEM) images, and the electron diffraction pattern in the selected area showed amorphous crystalline (**Figure 2E and S3B**). The stability of dCas9/CaP/PEI-PEG-bHb nanoparticles was also confirmed by DLS and western blot**.** The nanoparticles exhibited nearly unchanged size stability in solution for thirty days (**Figure S4A**), while the level of free-dCas9 proteins in the supernatant slightly increased after 10 days compared to the initial day (**Figure S4B**). These data suggest that the dCas9/CaP/PEI-PEG-bHb nanoparticle improves the efficiency of incorporating the dCas9 proteins with gRNA molecules and did not significantly aggregate or degrade over time.

### Evaluating the efficiency of the β-hydroxybutyric acid (bHb) in dCas9/CaP/PEI-PEG-bHb

**Figure 3:**
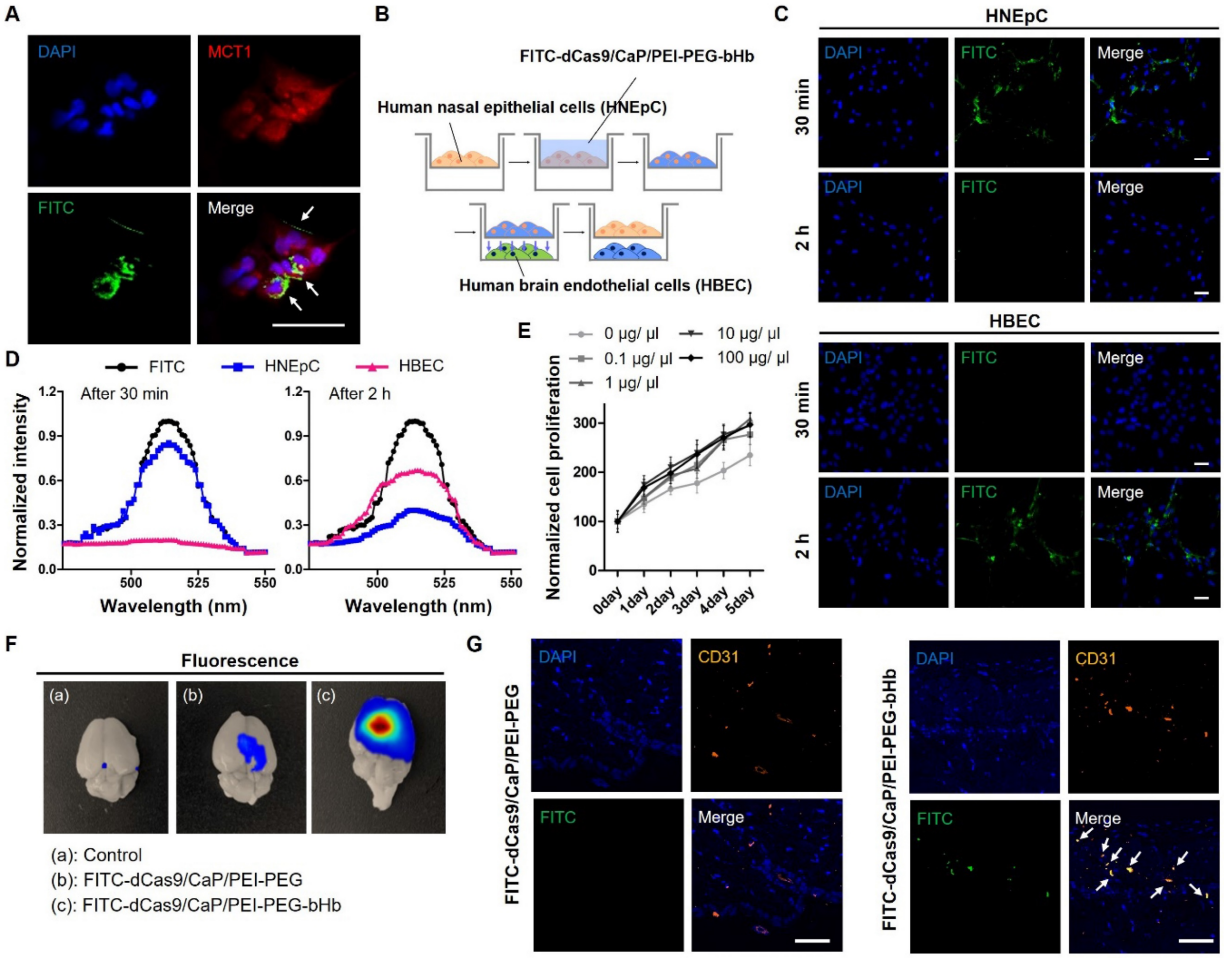
Effects of β-Hydroxybutyric acid (bHb) on dCas9/CaP/PEI-PEG-bHb *in vitro* and *in vivo*. **A** Confocal laser scanning microscopy (CLSM) images of HNEpCs treated with FITC-dCas9/CaP/PEI-PEG-bHb. Nuclei were stained with DAPI (blue), MCT1 was stained with red, and green signals represent FITC. **B** Schematic diagram of the indirect co-culture experiment. After the delivery of dCas9/CaP/PEI-PEG-bHb to human nasal epithelial cells (HNEpCs), the cells were transferred for co-culture with human brain endothelial cells (HBECs). **C** CLSM images of HNEpCs and HBECs taken at 30 mins and 2 h after delivery of FITC-dCas9/CaP/PEI-PEG-bHb. **D** Cellular uptake efficiencies determined by green fluorescence intensity of each cell. **E** Cell proliferation determined by WST-1 assay. Cells were treated with various concentrations of the nanoparticle solutions. Error bars indicate mean ± S.D. (*n* = 3). **F** Bioimaging of FITC-dCas9/CaP/PEI-PEG-bHb uptake in mouse brains 1 h after the intranasal delivery of FITC-incorporated nanoparticles with the dCas9-VP64 system. **G** Representative CLSM images of the brain tissues. Nuclei were stained with DAPI (blue), CD31 (endothelial cell marker) was stained with orange, and green signals represent FITC. (Scale bar: 50 μm)

To examine the effectiveness of β-hydroxybutyric acid (bHb), we tested whether the addition of bHb in dCas9/CaP/PEI-PEG-bHb makes it possible for the nanoparticle to be delivered intranasally and regulate the targeted gene in the brain *via* the dCas9-VP64 system (**Figure 1A**). To investigate the interaction of bHb in dCas9/CaP/PEI-PEG-bHb with monocarboxylate transporter 1 (MCT1) in human nasal epithelial cells (HNEpCs), the uptake of the dCas9/CaP/PEI-PEG-bHb in HNEpCs was evaluated by confocal laser-scanning microscopy (CLSM) (**Figure 3A**). For this experiment, fluorescein isothiocyanate (FITC) was loaded into the nanoparticles with the dCas9-VP64 system. MCT1 were stained with red, and green fluorescence from the FITC-dCas9/CaP/PEI-PEG-bHb indicated that their presence on the cell surface. We next evaluated the protein expression levels of MCT1 in HNEpCs and human brain endothelial cells (HBECs), comparing them to other cell lines (vascular smooth muscle cells (vSMCs), human breast cancer cells (BT474)) (**Figure S5A**). When vSMCs and BT474 were treated with the dCas9/CaP/PEI-PEG-bHb under the same conditions as HNEpCs and HBECs, dCas9 protein was not detected (**Figure S5B**). The expression of the MCT1 transporter was higher in HNEpCs and HBECs compared to other cell lines. We next investigated the uptake mechanism of the nanoparticles into cells using various metabolic inhibitors (MCT1 inhibitor (MCT1 receptor inhibitor), genistein (caveloine-dependent endocytosis inhibitor), chlorpromazine (clathrin-mediated endocytosis inhibitor), and cytochalasin B (macropinocytosis inhibitor)) (**Figure S6**). The nanoparticles were found to be internalized into the HNEpCs under genistein and cytochalasin B conditions. Conversely, dCas9 proteins were weakly detected after treatment with MCT1 inhibitor, chlropromazine, and incubation at 4°C. These results indicate that the uptake of dCas9/CaP/PEI-PEG-bHbs is mainly driven by transporter-mediated transcytosis, clathrin-mediated endocytosis, and energy-dependent endocytosis. The main factors for influencing the passive and/or carrier mediated delivery of particles from the nose to the brain are size and lipophilicity 61. The dCas9/CaP/PEI-PEG-bHb nanoparticles were confirmed to have a small size that is approximately 50 nm (**Figure 2A and 2E**). Therefore, we expected that the nanoparticles would mainly be transported through a transport-mediated mechanism in the olfactory bulb to the brain.

To evaluate whether HNEpCs uptake and transfer nanoparticles to HBECs, we treated HNEpCs with FITC-dCas9/CaP/PEI-PEG-bHb, and then the cells were transferred to 6-well plates with HBECs (**Figure 3B**). Green fluorescence from FITC was detected only in the HNEpCs 30 mins after the nanoparticles delivery (**Figure 3C**). In contrast, the green fluorescence signal was significantly observed in HBECs after 2 hours. The fluorescence intensity of each cell, indicative of FITC-dCas9/CaP/PEI-PEG-bHb uptake, reached a maximum of 85.8% in the HNEpC 30 min after delivery (**Figure 3D**). After 2 hours, the green fluorescence intensity increased to 66.4% in the HBECs.

The study on the cytotoxicity of dCas9/CaP/PEI-PEG-bHb is crucial for their application in mice. **Figure 3E** shows the effect of dCas9/CaP/PEI-PEG-bHb nanoparticles on the proliferation of HNEpCs. For all concentrations of NPs, no other effect on cell proliferation or differentiation was observed under cell culture conditions. We confirmed the gene editing capability of dCas9/CaP/PEI-PEG in HBECs after transferring the particles from HNEpCs treated with various nanoparticles (**Figure S7**). We examined the upregulation of SIRT1 in endothelial cells in particular as SIRT1 has been shown to be involved in endothelial angiogenesis after ischemia 62. mRNA and protein expression levels of SIRT1 were significantly increased in HBECs by HNEpCs treated with dCas9/CaP/PEI-PEG-bHb. These results suggest that the dCas9/CaP/PEI-PEG-bHb nanoparticle can bind to MCT1 transporter in nasal epithelial cells which facilitate its to transfer to brain endothelial cells.

### Induction of SIRT1 expression in the brain *via* intranasal administration of dCas9/CaP/PEI-PEG-bHb nanoparticles

We next asked whether our system of dCas9/CaP/PEI-PEG-bHb nanoparticles could be delivered to the brain *via* the nasal route, and be used to induce the expression of SIRT1 *in vivo*. For these experiments, we first administered the nanoparticles intranasally to mice in a supine position (**Figure S8**). We assessed the necessity of the bHb conjugation for the delivery of the nanoparticles to the brain. First, we observed fluorescent signals in the brains of FITC-dCas9/CaP/PEI-PEG-bHb-treated mice by optical imaging (**Figure 3F**). FITC was highly detected in the brain sections of dCas9/CaP/PEI-PEG-bHb-treated mice, in contrast to low detection in mice treated with dCas9/CaP/PEI-PEG nanoparticles without bHb (**Figure 3F**). The mRNA and protein levels of MCT1 expression are distributed through the entire mouse brain 63, 64. MCT1 expression is weakly observed in pericytes and astrocytes, and it had been observed that substances are absorbed through MCT1 more than 8-fold in brain endothelial cells than in pericytes and astrocytes 65, 66. While MCT1-positive glial-like cells were also visible in the mouse brain 67, 68, MCT4 as opposed to MCT1 has been reported as the glial monocarboxylate transporter 69. Furthermore, while MCT1 protein was prominently expressed in endothelial cells forming microvessels in the adult brain 70, the immunoreactivity of MCT1 in neurons was not observed in the adult mouse brain 71. These results suggest that our particles would primarily be taken up by endothelial cells through MCT1 in the brain. Therefore, to evaluate whether brain endothelial cells uptake dCas9/CaP/PEI-PEG in mice, we stained CD31 (an endothelial cell marker) in brain tissues after intranasal administration (**Figure 3G**). FITC within the nanoparticles was observed only in dCas9/CaP/PEI-PEG-bHb-treated mice, and overlapped with CD31. These data suggest that endothelial cells have taken up the dCas9/CaP/PEI-PEG-bHb in the brain. We also confirmed the presence of dCas9-VP64 proteins in the thalamus and cerebral cortex of the brain of mice that received intranasal administration of dCas9/CaP/PEI-PEG-bHb (**Figure S9A**). mRNA and protein expression levels of SIRT1 were increased in the brain after treatment with dCas9/CaP/PEI-PEG-bHb (**Figure S9B**). The target gene was dramatically upregulated *in vivo* (13-fold for Sirt1 on average). These results showed that the dCas9-VP64 system encapsulated in dCas9/CaP/PEI-PEG-bHb nanoparticles could effectively be delivered intranasally to the brain via bHb, and the protein-based dCas9-VP64 system could induce upregulation of the target gene in the brain.

### Effect of dCas9/CaP/PEI-PEG-bHb in a mouse model of ischemic stroke

**Figure 4:**
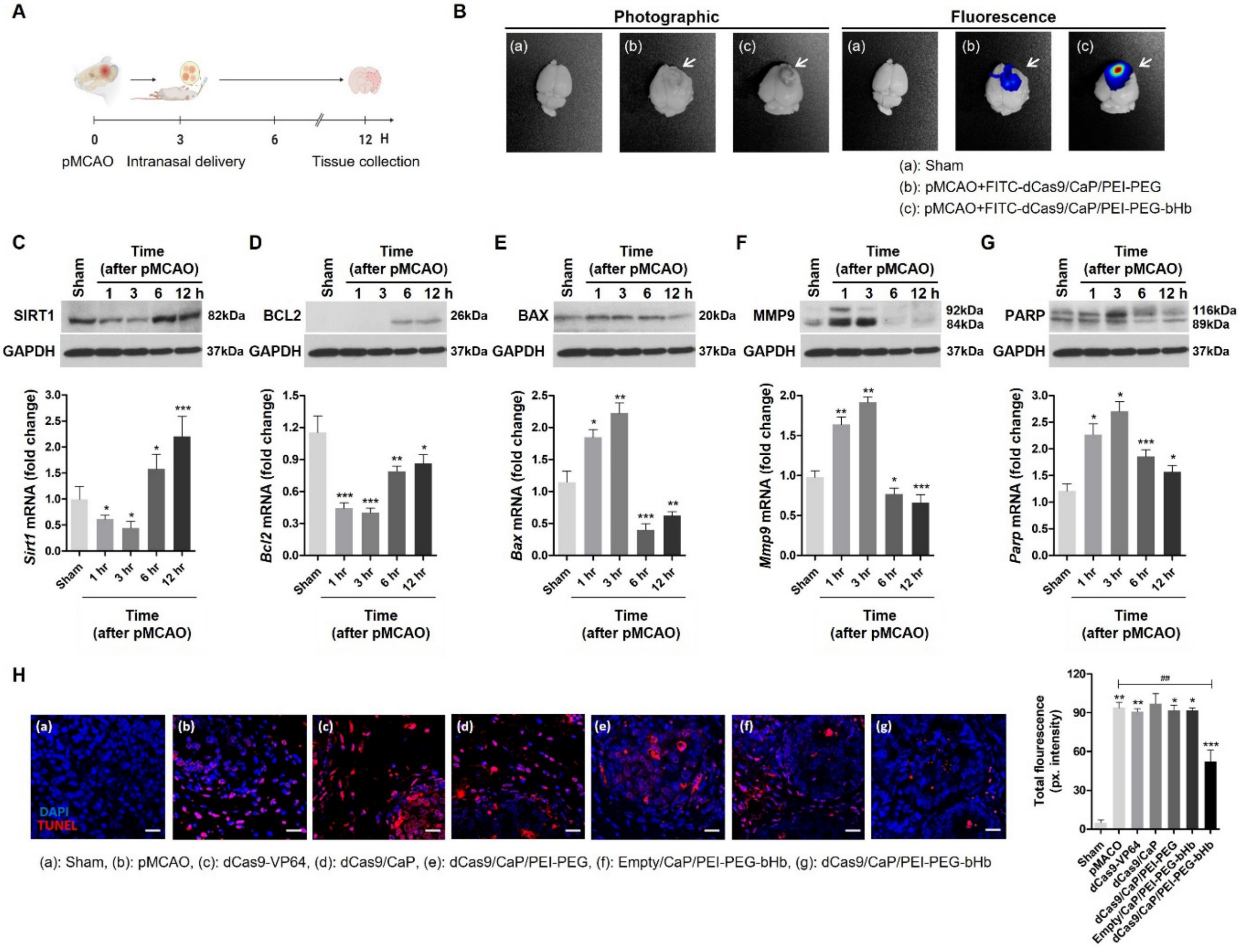
*In vivo* gene editing and therapeutic effects of dCas9/CaP/PEI-PEG-bHb on the ischemic brain. A Timeline of *in vivo* experiment. The particles were delivered intranasally at 3 hours after pMCAO, and tissues were harvested at 12 hours after pMCAO. Figure created with BioRender.com. B Bioimaging of FITC-dCas9/CaP/PEI-PEG-bHb uptake in mouse brains 1 h after the intranasal delivery of FITC-incorporated nanoparticles with the dCas9-VP64 system in pMCAO. C-G mRNA and protein expression levels of *Sirt1, Bcl2, Bax, Mmp9* and *Parp* in the ischemic brain after intranasal delivery of the nanoparticles at 3 hours after pMCAO. All data are expressed as fold change relative to the sham surgery group after normalization to *Gapdh*. *Mmp2 and Parp appear as two bands on the western blots for full length and cleaved proteins per the manufacturers' data.* Error bars indicate mean ± S.D. (*n* = 10 mice per group, *P < 0.05, **P < 0.01, ***P < 0.001 versus sham). H TUNEL staining of infarcted brain sections by confocal laser scanning microscopy. Brain tissues were stained with immunofluorescent TUNEL (red; apoptotic cells), and nuclei were stained with DAPI (blue) (Scale bar: 20 μm). Quantitative analysis of immunofluorescent TUNEL using ImageJ. Error bars indicate mean ± S.D. (*n* = 3 mice per group, 3 sections per mouse, *P < 0.05, **P < 0.01, ***P < 0.001 versus sham surgery, ^##^*P* < 0.01 versus pMCAO).

To demonstrate a potential therapeutic application of dCas9/CaP/PEI-PEG-bHb in the brain, we asked whether our system could ameliorate a mouse model of ischemic stroke. We used the permanent middle cerebral artery occlusion (pMCAO) model, an ideal model for human stroke (**Figure S10A**). Ischemic damage after pMCAO is more severe than a transient middle cerebral artery occlusion (tMCAO), and fatality in pMCAO during the 12 hours after successful pMCAO is high 72. Thus, we examined the pMCAO model within 6 hours after surgery for its clinical use in severe stroke. The area of ischemic damage after 6 hours of pMCAO is large (**Figure S10B**). We also confirmed decreases in mRNA expression levels of *Sirt1* at 1 h, 3 h, and 6 h after pMCAO (**Figure S10C**). *Sirt1*, silent information regulator 2 homologue 1, protects against cerebral ischemic injury, but expression of this gene is reduced by brain injury 73. Thus, we asked whether our system could be used to induce the expression of *Sirt1 in vivo* to treat pMCAO. We delivered the dCas9/CaP/PEI-PEG-bHb intranasally to the mice 3 hours after pMCAO (**Figure 4A**), and examined gene induction by the protein-based dCas9-VP64 system targeting *Sirt1*. We observed fluorescent signals in the brains of FITC-dCas9/CaP/PEI-PEG-bHb-treated mice by optical imaging in pMCAO (**Figure 4B**). FITC was highly detected in the infarcted brains of dCas9/CaP/PEI-PEG-bHb-treated pMCAO mice, in contrast to low detection in pMCAO mice treated with dCas9/CaP/PEI-PEG without bHb. We confirmed that the mRNA and protein expression levels of *Sirt1* increased after treatment with dCas9/CaP/PEI-PEG-bHb, even though the mice's MCAs were still occluded as part of the pMCAO (**Figure 4C**). To assess the therapeutic effects, we evaluated other genes correlated with *Sirt1*. The downregulation of anti-apoptotic *Bcl2* and upregulation of pro-apoptotic *Bax* have been associated with cell death in ischemic stroke 74, 75. In fact, decreasing the infarct area following ischemia is affected by the overexpression of *Bcl2* 76. *Mmp9* is related to neuroprotection, inflammation, and apoptosis, and is upregulated after cerebral ischemia, where it is associated with increasing matrix degradation and disrupting the blood-brain barrier 77. It has been reported that the overexpression of *Parp* attenuated inflammation and cell survival, and the inhibition of *Parp* could protect the brain against acute ischemic stroke 78. We therefore investigated whether the administration of dCas9/CaP/PEI-PEG-bHb was correlated with the regulation of these genes. pMCAO decreased mRNA expression levels of *Bcl2*, and increased mRNA expression levels of *Bax*, *Mmp9* and *Parp* (**Figure S11**). However, the dCas9-VP64 system prevented *Bcl2* downregulation when the stroke induced mice were treated with dCas9/CaP/PEI-PEG-bHb (**Figure 4D**). Conversely, expression levels of *Bax*, *Mmp9* and *Parp* were decreased in the mice after intranasal delivery of the dCas9/CaP/PEI-PEG-bHb (**Figure 4E-G**). In addition, we confirmed mRNA expression levels of *Bcl2*, *Bax*, *Mmp9* and *Parp* in the ischemic brain at 6 h after pMCAO using intranasal delivery of various prepared nanoparticles such as free dCas9, dCas9/CaP, dCas9/CaP/PEI-PEG, and empty/CaP/PEI-PEG-bHb (without the dCas9 system) (**Figure S12**). We found that these mRNA levels were expressed similarly to mRNA levels observed in the ischemic brain. Although the size of the infarcted area could not be reduced as the mice already had severe ischemic damage by 3 hours as evidenced by the changes in *Bcl, Bax, Mmp9* and *Parp*, survival time in mice treated with dCas9/CaP/PEI-PEG-bHb was significantly longer than pMCAO alone. Specifically, 100% (7/7) of mice with pMCAO did not survive to 24 hours with 71% (5/7) not surviving to 12 hours, whereas 80% of mice treated with dCas9/CaP/PEI-PEG-bHb survived to 24 hours (*p*=0.02). To determine the effect of the dCas9/CaP/PEI-PEG-bHb on brain edema in pMCAO, we assessed the brain water content in infarcted brains following the nanoparticle treatment. It was significantly decreased in mice treated with the dCas9/CaP/PEI-PEG-bHb compared with pMCAO without nanoparticles and pMCAO with dCas9/CaP/PEI-PEG without bHb (**Figure S13**). In addition, we confirmed that dCas9/CaP/PEI-PEG-bHb regulated genes were associated with apoptosis. We investigated apoptotic cells in the infarcted brain regions using the TUNEL assay (**Figure 4H**). The number of apoptotic cells in the brains that received dCas9/CaP/PEI-PEG-bHb was significantly decreased compared to other nanoparticles. Overall, our results suggest that our system can efficiently induce *Sirt1*, and *Sirt1* can regulate other neuroprotective genes to ameliorate the secondary effects of ischemic stroke such as edema.

### *In vivo* toxicity of the dCas9/CaP/PEI-PEG-bHb

As a final step toward demonstrating the potential therapeutic application of the dCas9/CaP/PEI-PEG-bHb nanoparticle, we evaluated the safety and toxicity of dCas9/CaP/PEI-PEG-bHb *in vivo*. We first evaluated the efficiency of dCas9/CaP/PEI-PEG-bHb incorporation in various organs (**Figure S14A**). The target gene did not show any change in various organs of mice treated with different nanoparticles except for dCas9/CaP/PEI-PEG-bHb. The mRNA expression levels of *Sirt1* in the brain of mice treated with dCas9/CaP/PEI-PEG-bHb were the highest compared to other various organs, but *Sirt1* mRNA expression was also elevated 2-fold in the lungs. However, the mRNA expression levels of *Sirt1* returned to control levels 7 days after intranasal delivery of dCas9/CaP/PEI-PEG-bHb (**Figure S14B**). Also, body weight did not change during the 32 days after treatment, and different organs showed no morphological changes or signs of inflammation by hematoxylin and eosin (H&E) histology analysis 30 days after intranasal administration of the dCas9/CaP/PEI-PEG-bHb (**Figure S15 and S16**). These results suggest that the dCas9/CaP/PEI-PEG-bHb can be delivered efficiently and safely to the brain *in vivo*. In conclusion, our study here revealed that the protein-based dCas9-VP64 system encapsulated in dCas9/CaP/PEI-PEG-bHb could have a neuroprotective effect and be a potential therapeutic strategy for preventing the secondary effects of ischemic stroke.

## Discussion

The most devastating type of ischemic stroke is a large vessel occlusion which requires prompt treatment to restore blood flow and prevent further damage. However, reperfusion is often infeasible in a timely manner. We therefore developed an intranasal delivery system using nanoparticles conjugated to β-hydroxybutyric acid (bHb) to deliver the treatment directly and rapidly to the brain even in the presence of a large vessel occlusion. We also established a gene-editing tool for ischemic stroke therapy using a CRISPR/dCas9 system targeting *Sirt1* to protect the brain from the effects of ischemia. We demonstrated the effectiveness of this approach in achieving changes in gene expression in the ischemic brain, as well as, reducing cerebral edema and increasing survival. Our development of a delivery system using dCas9/CaP/PEI-PEG-bHb nanoparticles for targeting the brain to treat ischemic stroke in the presence of a large vessel occlusion provides a promising new strategy for an efficient and easily deliverable targeted gene therapy in the ischemic brain that does not involve strict time restrictions or require specialized care.

Protein-based CRISPR gene therapy shows great promise for treating neurological disorders but faces major delivery challenges in the brain, especially in areas affected by stroke. Stroke due to large artery occlusion makes it difficult to deliver the protein-based CRISPR proteins intravenously, and can cause off-target effects due to reduced blood flow to the affected area and inflammation. The intranasal delivery system in this study is designed to overcome the BBB. However, further advancements in protein engineering and non-viral delivery systems are needed to further optimize our approach.

Dead Cas9 (dCas9) is a variant of Cas9 that suppresses target gene expression without DNA double strand breaks 79, 80. The off-target effects of dCas9 are less than a Cas9 system; however, they still pose a potential risk. Mitigating off-target activity is crucial for the practical application of CRISPR-mediated gene therapy. One approach to address off-target effects is to use lipid nanoparticles to deliver and protect single-guide RNAs (sgRNAs) by preventing the disruption of their secondary structure. In our study, we utilized lipid nanoparticles to protect sgRNAs and employed a protein-based dCas9 system 81.

Previous studies have explored various nanoparticle-based approaches for intranasal delivery to the brain, including nanoemulsions, chitosan-based nanoparticles, and polymeric nanoparticles. Nanoemulsions have been investigated for their ability to enhance drug solubility and absorption through the nasal mucosa, and can effectively encapsulate lipophilic drugs and improve their bioavailability 82, 83. However, they often require surfactants and co-surfactants, which can cause mucosal irritation and limit their long-term use for chronic conditions. Chitosan-based nanoparticles offer biocompatibility and mucoadhesive properties, which enhance their retention time in the nasal cavity and facilitate drug absorption 84, 85. Chitosan's positive charge allows it to open tight junctions between epithelial cells, promoting paracellular transport 86. Despite these advantages, chitosan nanoparticles may have limited stability in physiological conditions, and their positive charge can lead to rapid clearance from the nasal cavity due to mucociliary clearance mechanisms. Polymeric nanoparticles, such as those made from PLGA (poly(lactic-co-glycolic acid)), provide controlled and sustained release of therapeutic agents 45, 87. They are biodegradable and can be engineered to protect sensitive molecules from degradation. However, the complexity of their fabrication process and potential issues with scaling up production can be significant drawbacks. In comparison, the dCas9/CaP/PEI-PEG-bHb nanoparticle system proposed in this study combines the strengths of these approaches while addressing some of their limitations. The use of calcium phosphate nanoparticles ensures stability and protection of the CRISPR/dCas9 system against degradation. Conjugation with β-hydroxybutyrates (bHb) targets MCT1, facilitating efficient transport across the nasal epithelium into the brain. Furthermore, the small size and surface charge of our nanoparticles help with delivery efficacy and intracellular trafficking in nasal epithelial cells. Additionally, the polyethyleneimine-polyethylene glycol (PEI-PEG) coating enhances biocompatibility and reduces potential immunogenicity. Our system specifically targets neuroprotective genes, such as *SIRT1*, providing a tailored therapeutic approach for ischemic stroke. This targeted gene therapy offers potential benefits over conventional drug delivery systems by directly modulating gene expression to confer neuroprotection and promote recovery.

Future research is required to optimize the delivery efficiency and targeting specificity of the intranasal CRISPR/dCas9 system. Enhancing the nanoparticle formulation to increase the stability and bioavailability of the therapeutic agents is a key area for improvement. This includes refining the composition of the CaP nanoparticles and exploring alternative conjugation strategies to maximize interaction with nasal epithelial cells and subsequent transport to the brain. Additional functional outcome studies and histological analyses are needed to provide a more holistic evaluation of the therapeutic efficacy of our approach. Long-term safety and efficacy studies are essential to ensure that repeated administration of the nanoparticles does not induce adverse effects or immune responses. Investigating the potential for cumulative effects or long-term gene expression changes will be crucial for translating this therapy to clinical settings. Expanding the scope of target genes beyond *SIRT1* to include other neuroprotective or regenerative genes could further enhance the therapeutic potential of this approach. Identifying and validating additional gene targets that contribute to neuroprotection and recovery post-stroke can provide a more comprehensive treatment strategy.

Clinical trials will be necessary to establish the translational applicability of this therapy in humans. These trials should aim to determine the optimal dosing regimen, evaluate the therapeutic window, and assess the overall clinical benefits in stroke patients. Integrating this gene therapy approach with existing stroke treatments, such as thrombolytics or mechanical thrombectomy, could provide synergistic effects and improve patient outcomes. Exploring combinatory therapies may offer a more robust and effective treatment paradigm for acute ischemic stroke.

In conclusion, we have developed a protein-based CRISPR/dCas9 nanoparticle system that can be efficiently delivered to the brain intranasally. The CRISPR/dCas9 is prevented from degradation by calcium phosphate encapsulation and intranasal delivery is facilitated by conjugation with β-hydroxybutyrate. We have demonstrated that intranasal delivery of the dCas9/CaP/PEI-PEG-bHb nanoparticles in mice that underwent permanent middle cerebral artery occlusion induces significant upregulation of the target gene, *Sirt1*, decreased brain edema, and increased survival with no significant *in vivo* toxicity. These results show that the dCas9/CaP/PEI-PEG-bHb nanoparticles may serve as a potential novel delivery system for gene therapy in the treatment of acute ischemic stroke due to large vessel occlusion.

## Supplementary Material

Supplementary figures.
