# Supplementary material for: Brain-targeted intranasal delivery of protein-based gene therapy for treatment of ischemic stroke
Source: Theranostics. 2024 Aug 12;14(12):4773–86. doi: 10.7150/thno.98088 (PMC11373627; doi:10.7150/thno.98088)
Supplement: Supplementary file 1 — Supplementary figures. [file thnov14p4773s1.pdf]

SUPPLEMENTAL MATERIALS

Brain-targeted Intranasal Delivery of Protein-based Gene Therapy for Treatment of Ischemic Stroke

Jee-Yeon Ryu[1], Christian Cerecedo-Lopez[1,2], Hongkuan Yang[1,3], Ilhwan Ryu[4,5*] and Rose Du[1*]

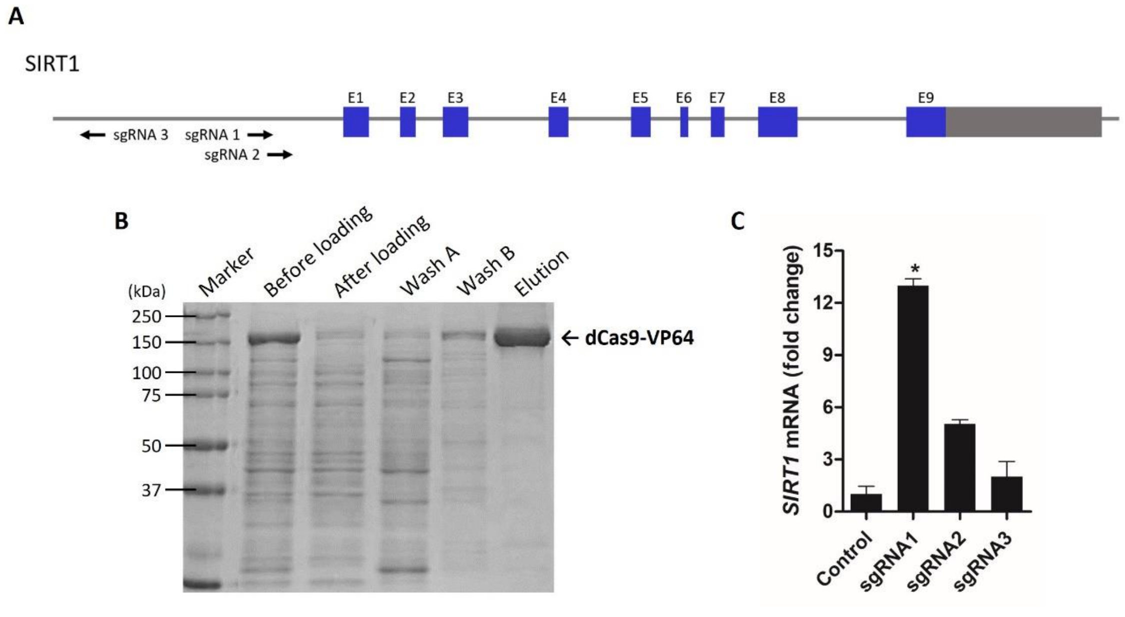

Figure S1. Screening of sgRNAs that guide dCas9-VP64 to activate *SIRT1* promoter and preparation of dCas9-VP64 protein. **A** Designed sgRNAs with different sequences. **B** SDS-PAGE analysis of dCas9-VP64 proteins. **C** mRNA expression levels of *SIRT1* after the delivery of sgRNA 1, 2 and 3 with dCas9-VP64 proteins in HBECs. Data is expressed as fold change relative to the control after normalization to GAPDH. Error bars indicate mean ± S.D. ($n = 3$, $^*P < 0.05$ versus control).

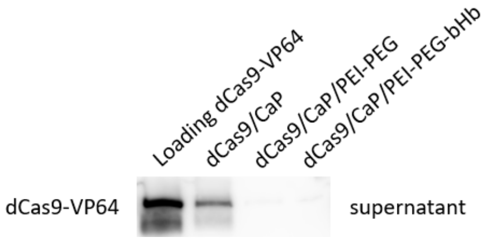

Figure S2. The encapsulation efficiency of the dCas9-VP64 proteins by western blot. The dCas9-VP64 proteins were obtained from the supernatant after centrifugation under different synthesis conditions of the nanoparticles. Sample loading was 20 μl per well.

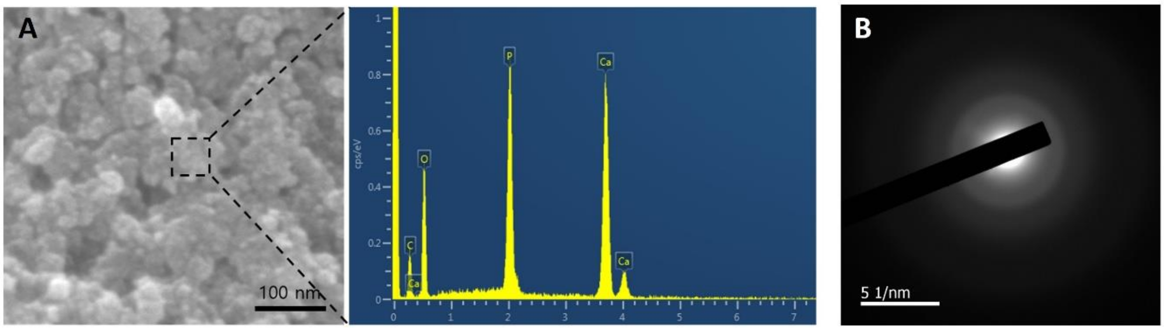

Figure S3. A The FE-SEM image and SEM-energy dispersive X-ray spectroscopy (EDX) analysis of the CaP nanoparticles. **B** The electron diffraction pattern of the dCas9/CaP/PEI-PEG-bHbs.

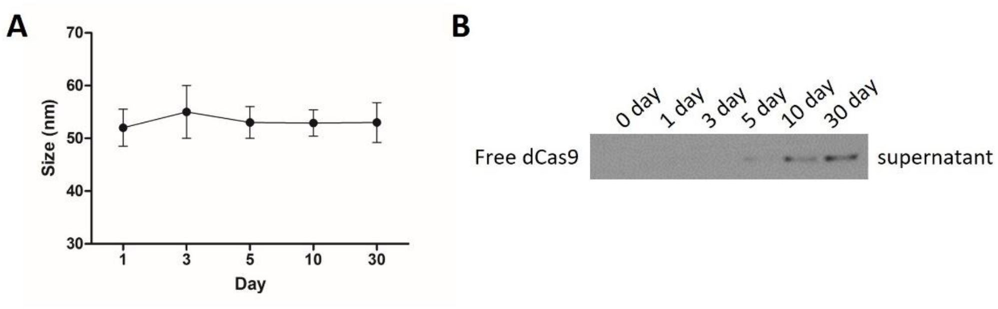

Figure S4. A The stability of the dCas9/CaP/PEI-PEG-bHbs was measured by dynamic light scattering (DLS). **B** The release pattern of free dCas9 proteins from the nanoparticles was evaluated by western blot. The dCas9 proteins were obtained from the supernatant at various time points, including 0, 1, 3, 5, 10, and 30 days after synthesis.

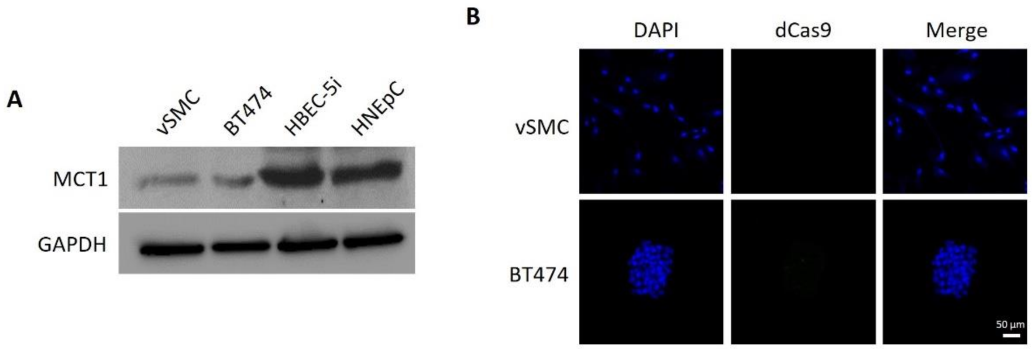

Figure S5. A Protein expression levels of monocarboxylate transporter 1 (MCT1) in various cell lines was confirmed by western blot. **B** After treating the dCas9/CaP/PEI-PEG-bHb in various cell lines, the cells were stained with anti-dCas9 (green), and the nuclei were stained with DAPI (blue).

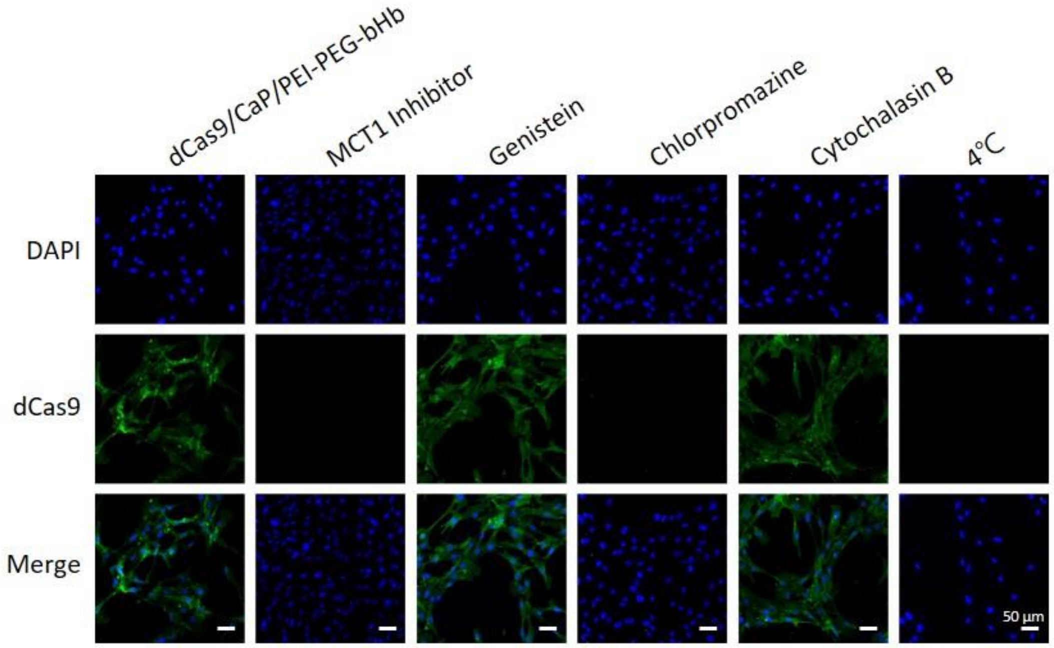

Figure S6. Uptake mechanism of the particles in HNEpCs. dCas9 protein was identified under treatment with various metabolic inhibitors (MCT1 inhibitor, genistein, chlorpromazine, and cytochalasin B) via confocal laser scanning microscopy (CLSM). The nuclei were stained with DAPI (blue) and cells were stained with anti-dCas9 (green).

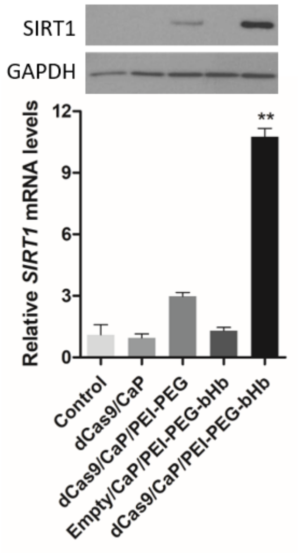

Figure S7. The gene editing capability of the dCas9/CaP/PEI-PEG-bHb *in vitro*. mRNA and protein expression levels of *SIRT1* in HBECs after transferring the particles from HNEpCs treated with various nanoparticles. Data is expressed as fold change relative to the control group after normalization to *GAPDH*. Error bars indicate mean ± S.D. (n = 3, $^{**}P < 0.01$ versus control).

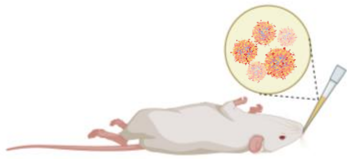

Figure S8. Schematic illustration of intranasal administration of the nanoparticles in mice.

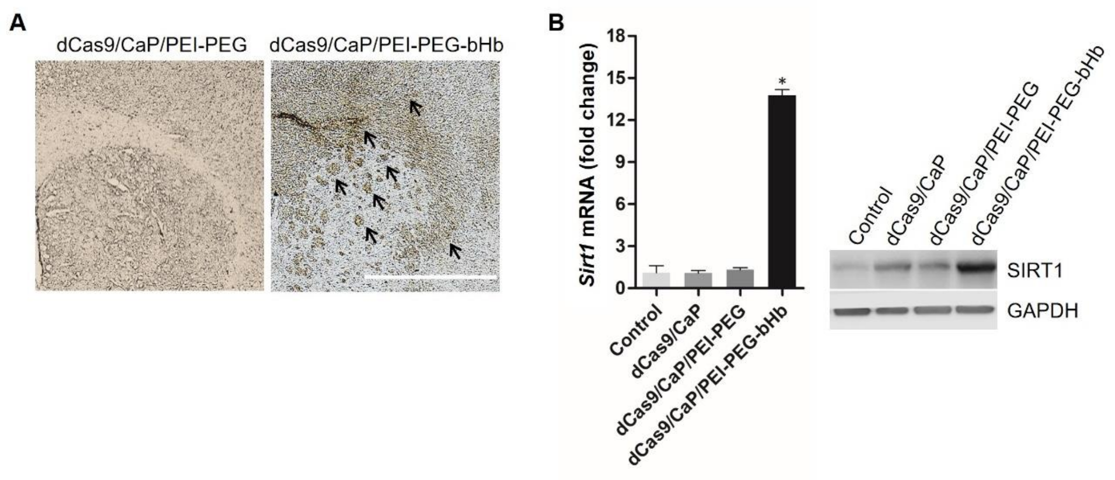

Figure S9. A Representative brain tissues stained for dCas9-VP64 proteins. **B** mRNA and protein expression levels of *Sirt1* in the brain after intranasal administration of the various nanoparticles. Data is expressed as fold change relative to the control group after normalization to *Gapdh*. Error bars indicate mean ± S.D. (n = 10 mice per group, *$P < 0.05$ versus control).

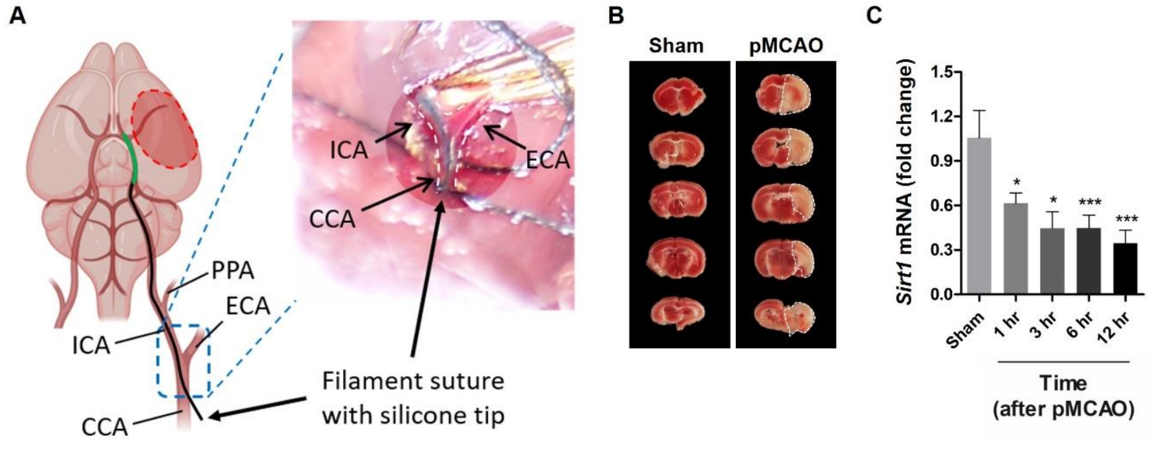

Figure S10. Establishment of a permanent middle cerebral artery occlusion (pMCAO) model for studying ischemic stroke. **A** Procedure for pMCAO surgery. The CCA, ICA, and ECA are exposed, and a silicone filament is inserted into the CCA and ICA until it reaches the MCA (see the Materials and Methods for details). Figure created with BioRender.com. **B** Representative photographs of TTC stained brain. The white areas represent the infarcted areas in pMCAO. **C** mRNA expression levels of *Sirt1* in the ischemic brain at 1, 3, and 6 hours after pMCAO. Data is expressed as fold change relative to the sham group after normalization to *Gapdh.* Error bars indicate mean ± S.D. (n = 3) (n = 10 mice per group, *$P < 0.05$, *** $P < 0.001$ versus sham). Abbreviations: CCA, common carotid artery; ICA, internal carotid artery; ECA, external carotid artery; MCA, middle cerebral artery; TTC, 2,3,5-triphenyltetrazolium chloride.

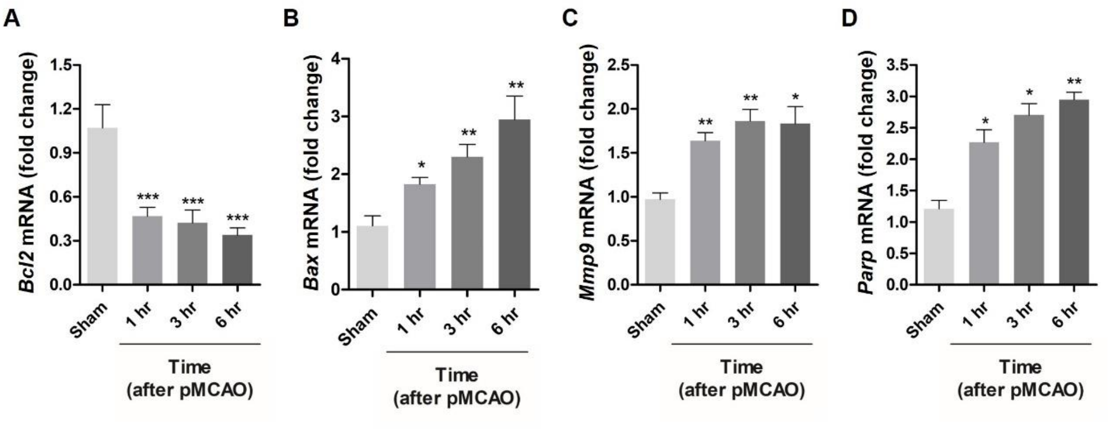

Figure S11. mRNA expression levels of *Bcl2, Bax, Mmm9* and *Parp* in the ischemic brain without nanoparticles. All data are expressed as fold change relative to the sham group after normalization to *Gapdh*. Error bars indicate mean ± S.D. (n = 10 mice per group, *$P < 0.05$, *** $P < 0.001$ versus sham).

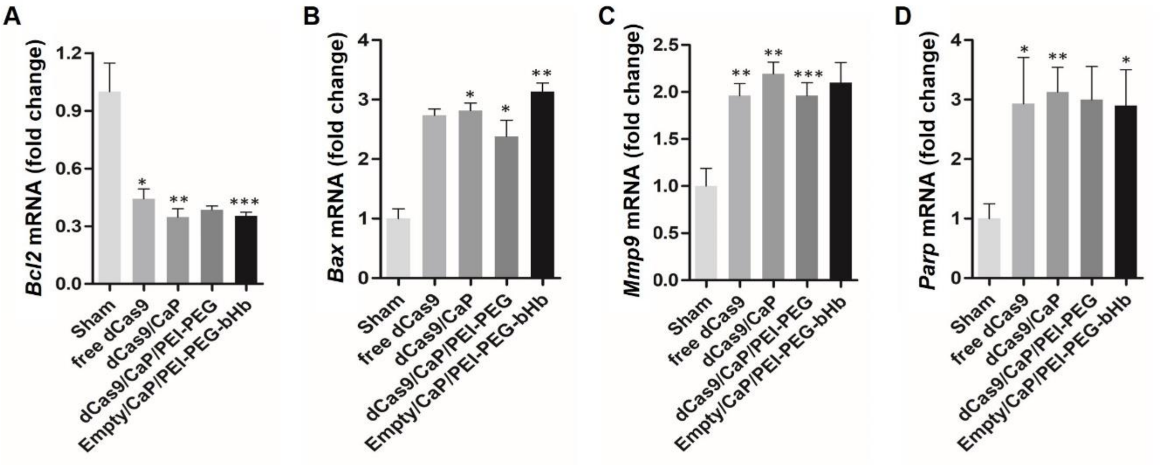

Figure S12. mRNA expression levels of *Bcl2, Bax, Mmp9* and *Parp* in the ischemic brain 6 h after pMCAO with the various prepared nanoparticles compared to sham surgery. All data are expressed as fold change relative to the sham group after normalization to *Gapdh*. Error bars indicate mean ± S.D. (n = 10 mice per group, *$P < 0.05$, **$P < 0.01$, *** $P < 0.001$ versus sham).

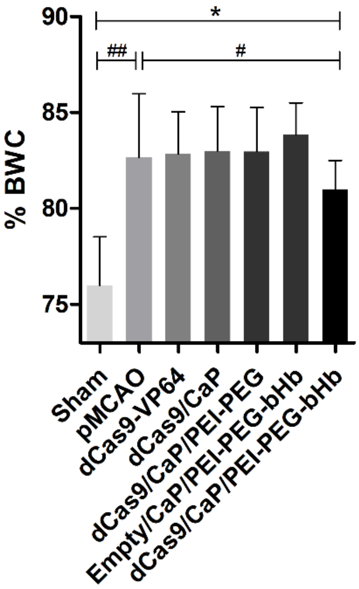

Figure S13. Assessment the effect of the nanoparticles on brain swelling 12 hours after pMCAO. Quantitative analysis of brain water content in the ischemic brain after intranasal delivery of the nanoparticles 3 hours after pMCAO. Error bars indicate mean ± S.D. (n = 3 mice per group, *$P < 0.05$ versus sham, $^{\#}P < 0.05$, $^{\#\#}P < 0.01$ versus pMCAO). (BWC, brain water content).

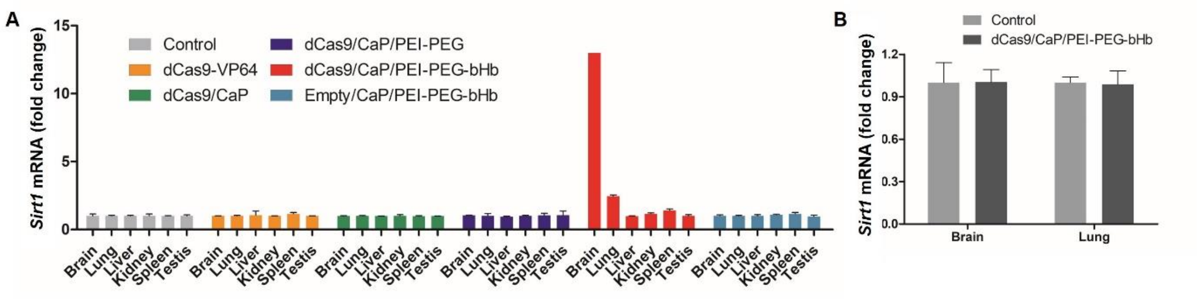

Figure S14. Distribution of the nanoparticles and toxicity *in vivo*. **A** mRNA and protein expression levels of *Sirt1* in various organs after intranasal administration of the various nanoparticles. **B** mRNA expression levels of *Sirt1* in the brain and lung at 7 days after intranasal delivery of the dCas9/CaP/PEI-PEG-bHb. All data are expressed as fold change relative to the control group after normalization to *Gapdh*. Error bars indicate mean ± S.D. (*n* = 10 mice per group). Abbreviations: Empty/CaP/PEI-PEG-bHb, CaP/PEI-PEG-bHb particle that does not contain the dCas9-VP64 protein with sgRNA.

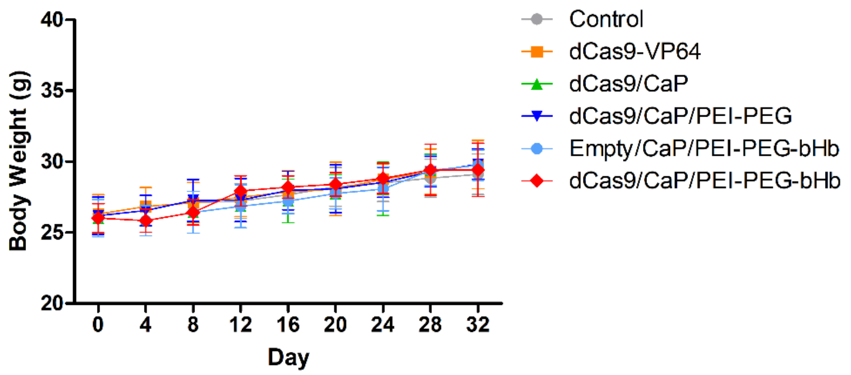

Figure S15. Body weight was measured every 4 days after initial treatment. Abbreviations: Empty/CaP/PEI-PEG-bHb, CaP/PEI-PEG-bHb particle that does not contain the dCas9-VP64 protein with sgRNA.

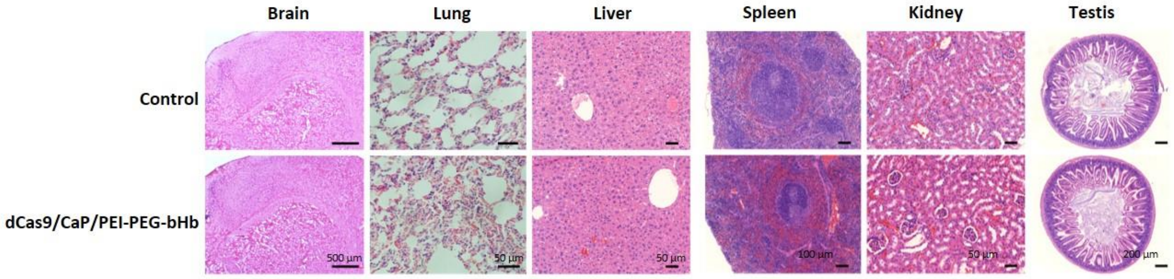

Figure S16. H&E staining of various organs from mice not treated and treated with dCas9/CaP/PEI-PEG-bHb.
